# Filamins Regulate Cell Spreading and Initiation of Cell Migration

**DOI:** 10.1371/journal.pone.0007830

**Published:** 2009-11-13

**Authors:** Massimiliano Baldassarre, Ziba Razinia, Clara F. Burande, Isabelle Lamsoul, Pierre G. Lutz, David A. Calderwood

**Affiliations:** 1 Department of Pharmacology and Interdepartmental Program in Vascular Biology and Therapeutics, Yale University School of Medicine, New Haven, Connecticut, United States of America; 2 Department of Cell Biology, Yale University School of Medicine, New Haven, Connecticut, United States of America; 3 Centre National de la Recherche Scientifique, Institut de Pharmacologie et de Biologie Structurale, Toulouse, France; 4 Université de Toulouse, Université Paul Sabatier, Institut de Pharmacologie et de Biologie Structurale, Toulouse, France; Dresden University of Technology, Germany

## Abstract

Mammalian filamins (FLNs) are a family of three large actin-binding proteins. FLNa, the founding member of the family, was implicated in migration by cell biological analyses and the identification of *FLNA* mutations in the neuronal migration disorder periventricular heterotopia. However, recent knockout studies have questioned the relevance of FLNa to cell migration. Here we have used shRNA-mediated knockdown of FLNa, FLNb or FLNa and FLNb, or, alternatively, acute proteasomal degradation of all three FLNs, to generate FLN-deficient cells and assess their ability to migrate. We report that loss of FLNa or FLNb has little effect on migration but that knockdown of FLNa and FLNb, or proteolysis of all three FLNs, impairs migration. The observed defect is primarily a deficiency in initiation of motility rather than a problem with maintenance of locomotion speed. FLN-deficient cells are also impaired in spreading. Re-expression of full length FLNa, but not re-expression of a mutated FLNa lacking immunoglobulin domains 19 to 21, reverts both the spreading and the inhibition of initiation of migration.

Our results establish a role for FLNs in cell migration and spreading and suggest that compensation by other FLNs may mask phenotypes in single knockout or knockdown cells. We propose that interactions between FLNs and transmembrane or signalling proteins, mediated at least in part by immunoglobulin domains 19 to 21 are important for both cell spreading and initiation of migration.

## Introduction

Cell migration is critical throughout development and in adulthood. Migration is required in response to injury or infection and excessive or impaired migration leads to pathologies ranging from brain defects, to vascular disease, inflammation and cancer [1]. Understanding cell migration is therefore of profound physiological and medical significance. Detailed analyses of cultured cells continue to provide insights into cell migration, permitting recognition of general principles and identification of key mechanisms and proteins [2].

Cell migration is an actin-dependent process and many proteins that regulate F-actin polymerization, de-polymerization, branching, cross-linking or bundling have now been implicated in controlling migration [3]. Filamins (FLNs) make up one important class of actin-binding and cross-linking proteins. Vertebrate FLNs are non-covalent dimers of 240–280 kDa subunits composed of an N-terminal actin-binding domain followed by 24 tandem immunoglobulin-like domains (IgFLN1–24), the last of which mediates dimerization [4]–[7]. Hinges between IgFLN15 & 16 (H1) and IgFLN23 & 24 (H2) result in a V-shaped flexible actin-crosslinker capable of stabilizing orthogonal networks with high-angle F-actin branching [8]. In addition, FLNs bind many transmembrane receptors, signaling and adapter proteins [5], [9], [10]. Through these interactions, often mediated by IgFLN16–24, FLNs complex multiple partners in close proximity to one another, potentially enhancing signal transduction by aiding assembly of networks linking receptors with signaling proteins and the cytoskeleton [5].

Humans have three *FLN* genes, encoding filamin A (FLNa, ABP-280 or filamin-1 [4]), filamin B (FLNb, ABP-278/276, β filamin or filamin-3 [11], [12]) and filamin C (FLNc, γ-filamin, ABPL or filamin-2 [13], [14]). With the exception of the H1 and H2 regions, and an 81 amino acid insertion in IgFLNc20, they show homology over their entire length. FLNa is the most abundant and widely expressed, FLNb is also widely expressed while FLNc is thought to be largely restricted to striated muscle [5], [6].

A requirement for FLNa during cell migration was first proposed based on the impaired locomotion of human melanoma lines lacking FLNa, and the ability of re-expressed FLNa to restore migration [15]. The *FLNA* gene is located on the X-chromosome and mutations leading to loss of FLNa expression or function were later identified as causative in X-linked periventricular heterotopia (PVH) in heterozygous females, revealing a role for FLNa in neuronal migration [16]. Furthermore, FILIP, a FLNa-interacting protein, was reported to control neuronal migration by regulating FLNa levels [17], [18]. Thus it was proposed that FLNa plays an essential role in the basic processes of cell migration. However, the phenotypes of two independently generated strains of FLNa-deficient mice and the observation that cells derived from these mice lacked obvious defects in migration [19], [20] has cast doubt on this conclusion. Furthermore, there is no evidence that the neurons in human PVH nodules lack FLNa, and, the percentage of heterotopic neurons is usually small despite the expectation that, assuming random X-inactivation, ∼50% of neurons in the heterozygous PVH patients should lack FLNa [9]. In addition, several males with FLNa mutations have PVH patterns similar to females [9]; while these are likely to be only partial loss-of-function mutations they indicate that most neurons organize correctly without any fully-functional FLNa. Nonetheless, other evidence continues to point to roles for FLNs in cell migration: over-expressed FLNa inhibits migration of M2 cells [15] and mouse cortical neurons [21], MEKK4^−/−^ mice exhibit PVH associated with neurons that over-express FLNa and b [21], and a male patient with severe PVH has a FLNa gene duplication [22], suggesting that excess FLNa leads to migration defects. Furthermore tight binding of FLN to integrin β tails correlates with inhibition of integrin-mediated cell migration [23]. *In vivo* migration defects have not been reported in FLNb^−/−^ mice but migration is impaired in FLNb^−/−^ fibroblasts, which also have reduced FLNa levels [24]. Thus, while FLNa may not be essential for cell migration, FLNs do appear to modulate migration and the possibility that FLNb may compensate for loss of FLNa has been raised [25]. Here we have used short-hairpin RNA (shRNA)-mediated knockdown of FLNa and/or FLNb, as well as acute proteasome-mediated degradation of all FLNs, to investigate the roles of FLN in cell migration. We find that cells deficient in FLN exhibit impaired initiation of random cell migration and defects in cell spreading but that once migration has been initiated FLN-deficient cells are capable of efficient migration at speeds comparable to wild-type cells. We suggest that the lack of overt migration phenotypes in many FLNa-deficient cells and organisms is likely to be due to the presence of co-expressed FLNb, or FLNc, which is able to compensate for the lack of FLNa, and that in some cases the plasticity of tissue development may accommodate the delay in initiation of cell migration caused by loss of FLNa.

## Results

### Generation of FLNa knockdown cells

**Figure 1:**
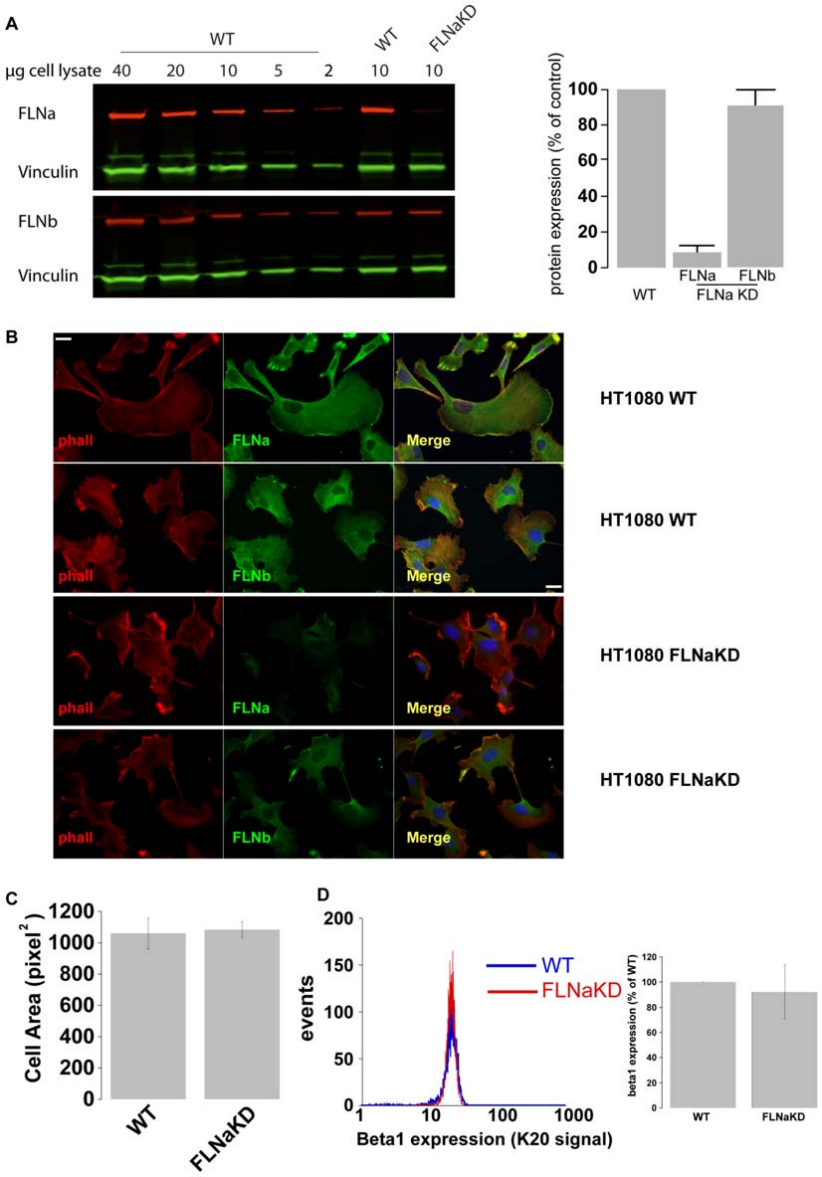
Generation of FLNaKD cells. **A**) FLNa and FLNb expression in FLNaKD cells was quantified comparing 10 µg of cell lysate to a curve of cell lysates prepared from HT1080 WT cells. Vinculin expression was used as loading control. The bar chart depicts average of 4 independent western blots normalized to control ± SEM. **B**) HT1080 WT or FLNaKD were plated on 5 µg/ml FN coated coverslips overnight, fixed, permeabilized and stained with anti-FLNa (FLNa), anti-FLNb (FLNb), phalloidin (phall) and DAPI (shown in blue in the merged image). Bar = 20 µm. **C**) HT1080 WT and HT1080 FLNaKD cells were detached, mixed and seeded on a 3.5 cm petri dish coated with 5 µg/ml FN, allowed to adhere and spread. Cells were fixed after 1 hour and stained with anti-FLNa to discriminate between WT and FLNaKD cells. The area was measured by manually rendering the cell contour in the phase images. 1 pixel^2^ = 1.664 µm^2^ (Sample size WT = 74; FLNaKD = 82 from 5 independent experiments). Error bars show SEM. **D**) Integrin beta1 present on the membrane surface of WT and FLNaKD cells. The left panel show histogram a representative histogram plot, the right panel shows the average of 4 experiments ± standard deviation.

To study the functions of FLNa we established HT-1080 human fibrosarcoma cell lines lacking FLNa expression. HT1080 cells were transfected with a pSM2c vector encoding shRNA against human FLNa, pools of stably transfected cells were obtained by antibiotic selection, and FLNa levels were assessed by western blotting. Two different shRNA sequences were tested and the one producing most efficient FLNa knockdown was chosen for further analysis. Clonal knockdown lines were obtained by limiting dilution. From these a FLNaKD line, with less than 10% of FLNa remaining compared to HT1080 wild type (WT) cells, was selected for further analysis (Figure 1A). Notably, FLNb, vinculin, actin and β1 integrin levels were unchanged in these FLNaKD cells (Figure 1A, B, D).

### FLNa knockdown cells can spread and form actin stress fibres

Previous studies have shown FLNa to play a critical role in maintaining mechanical stability of the cortical actin filaments [15], [26], so the effect of FLNa knockdown on the actin cytoskeleton was assessed by phalloidin staining. Even with an almost complete loss of FLNa, the actin cytoskeleton of HT1080 FLNaKD cells spread on fibronectin (FN) was not dramatically altered (Figure 1B). Furthermore, no significant effect on cell spreading was observed 1 hour after plating on 5 µg/ml FN (Figure 1C).

FLNa-deficient M2 melanoma cells exhibit a characteristic plasma membrane blebbing [15] but we did not observe blebbing in FLNaKD cells, either during routine cell culture or 1 hour after plating on FN (Figure 1B). The lack of membrane blebbing in FLNaKD HT1080 cells is consistent with observations in FLNa knockout fibroblasts [19], [20]. Analysis of another FLNaKD line and the polyclonal FLNaKD population (data not shown), confirmed our conclusion that significant reduction in FLNa levels does not dramatically alter HT1080 cell spreading, actin cytoskeleton or membrane stability.

### FLNa knockdown does not affect random cell migration

**Figure 2:**
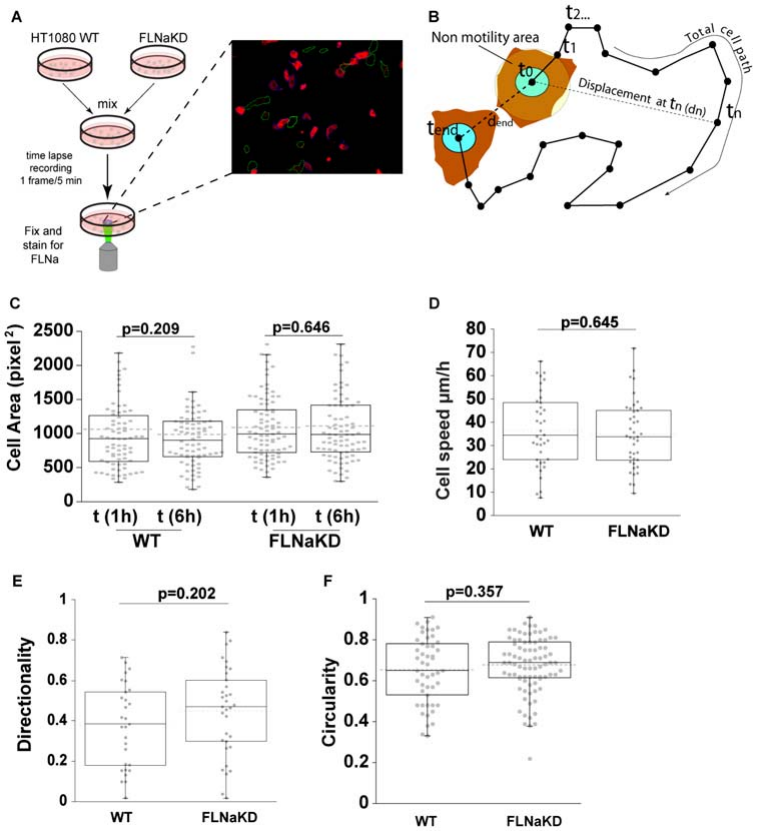
FLNaKD cells in random cell migration assay. **A**) Flow chart of the time-lapse experiments. Cells were suspended, washed, and control and knockdown cells mixed before seeding onto FN-coated plates. 10 minutes after plating unattached cells were washed away and imaging was initiated 1 hour after plating. At the end of the time-lapse recording, cells were fixed and stained for FLNa expression. WT cells are outlined in white, FLNaKD cells in green. **B**) Schematic diagram of migration parameters. Cell is considered migratory if max d>25 (average cell radius); final speed = Total cell path/t_end_; directionality  = d_end_/Total cell path. **C**) Comparison of cell area at the start and at the end of time-lapse recording. 1 square pixel^2^ = 1.664 µm^2^. Dots plot shows the overall population distribution (sample size: WT = 74; FLNaKD = 82; from 5 independent experiments), dotted line shows the mean, box and whiskers plots show quartiles. P values were calculated using a paired t-test. **D**) Speed of HT1080 WT and FLNaKD (sample size WT = 38; FLNaKD = 41; from 4 independent experiments). E) Directionality (defined as d_end_/total cell path) comparison between HT1080 WT and FLNaKD (sample size: WT = 29; FLNa = 33; from 3 independent experiments). F) Circularity (as defined in ImageJ = 4π(area/perimeter^2^) comparisons between HT1080 WT and FLNaKD cells 6 hours after plating (sample size: WT = 49; FLNaKD = 83; from 4 independent experiments).

To assess the role of FLNa in cell migration we used time-lapse microscopy to compare the random migration of FLNaKD and wild-type HT1080 cells. Cells were suspended, washed, and control and knockdown cells mixed before seeding onto FN-coated plates, 10 minutes after plating unattached cells were washed away and imaging was initiated 1 hour after plating. At the end of the time-lapse recording, cells were fixed and stained for FLNa expression allowing us to assess FLNa levels in the individual cells whose migration was recorded (Figure 2A and movie S1). By washing off unattached cells we synchronised the cell adhesion to within the initial 10 minute period, reducing variability due to differential attachment times. Furthermore, as both control and knockdown lines were seeded onto the same plate and imaged simultaneously, migration could be compared under the same conditions, and any correlation with the FLNa expression levels detected by immunofluorescence could be observed. This provides a control for potential variability in coating, temperature, serum, etc, between experiments and allows direct comparison of cell motility with FLNa levels. Overall, our migration protocol enables us to follow migration in detail in a controlled 2D environment over a defined time period (usually 5 hours), permitting measurement of the total distance traveled, the displacement from origin, and calculation of mean speed and directional persistence (Figure 2B). Additionally, the morphology and spreading of migrating cells were also assessed. Migration was generally recorded between 1 and 6 hours post plating as the cells were fully spread by 1 hour and the mean cell area did not increase during the time-lapse recording (Figure 2C; wild-type p = 0.209, 74 cells; FLNaKD p = 0.646, 82 cells from 5 independent experiments).

Using this approach we observed no significant difference in mean speed of the wild-type and FLNaKD cells (Figure 2D). The cells also showed no significant difference in directionality (Figure 2E) or in circularity (Figure 2F). Similar results were obtained with a second FLNaKD line (data not shown). This suggests that neither the motility nor spreading of HT1080 cells is impacted by FLNa knockdown.

### Generation and characterisation of FLNab double knockdown cells

**Figure 3:**
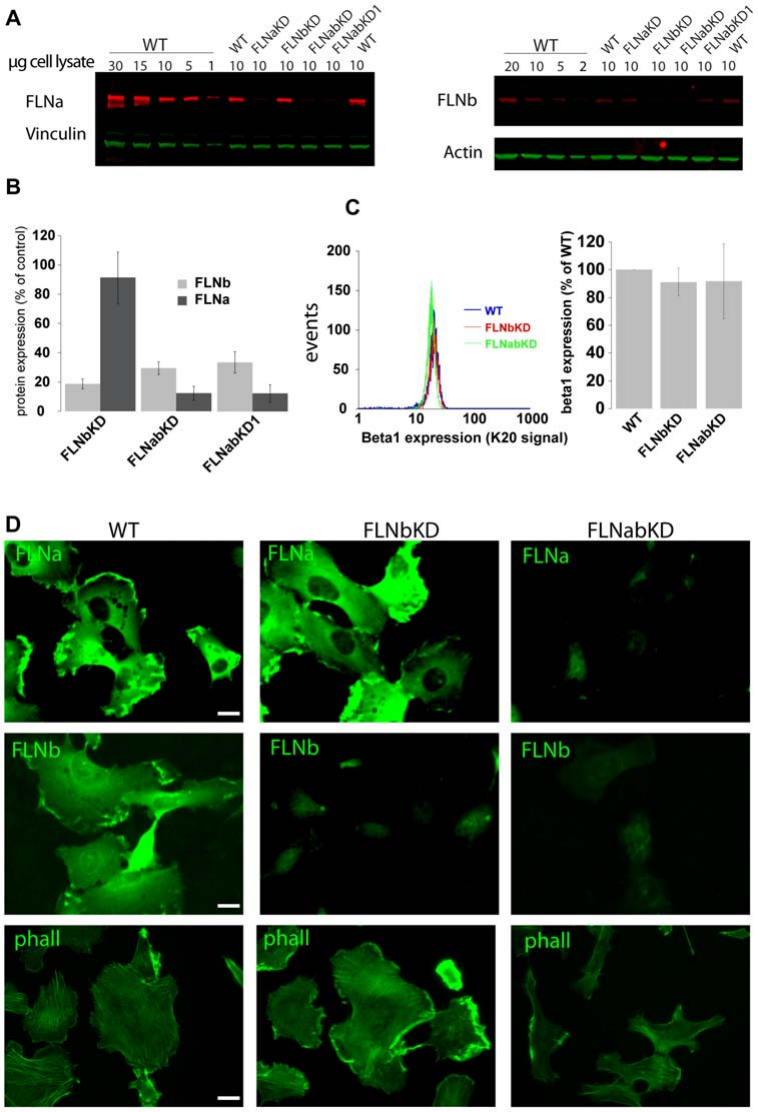
Generation of FLNabKD cells. **A**) FLNa and FLNb protein expression was quantified comparing 10 µg of cell lysate prepared from FLNaKD, FLNbKD and FLNabKD cells to a curve of cell lysates prepared from HT1080 WT lysates. Vinculin and actin were used as loading control. **B**) Quantification of FLNa and FLNb protein expression; bars show mean value normalized to control ±SEM (n = 4). **C**) Integrin beta1 present on the membrane surface of WT, FLNbKD and FLNabKD cells. The left panel shows a representative histogram plot, the right panel shows the average of 4 experiments ± standard deviation. **D**) HT1080WT, FLNbKD or FLNabKD cells were plated on FN-coated coverslips, incubated overnight, fixed, permeabilized and stained with anti-FLNa (FLNa), anti-FLNb (FLNb) or phalloidin (phall). Bar = 20 µm.

The preceding results, showing that wild-type levels of FLNa are not required for HT1080 cell migration, raise the possibility that endogenous FLNb (Figure 1A), may compensate for lack of FLNa [25]. To test this possibility we knocked down FLNb in HT1080 WT (FLNbKD) and FLNa knockdown (FLNabKD) cells. Cells were transfected with pGIPZ vectors encoding shRNA against human FLNb and pools of stably transfected cells were obtained by antibiotic selection. FLNb was significantly reduced in FLNbKD cells while FLNa was unaffected in these cells. Both FLNa and FLNb expression were reduced in the double FLNab KD cells (Figure 3 A,B). Vinculin, actin and surface β1 integrin levels were not significantly altered in these cells (Figure 3A and 3C).

**Figure 4:**
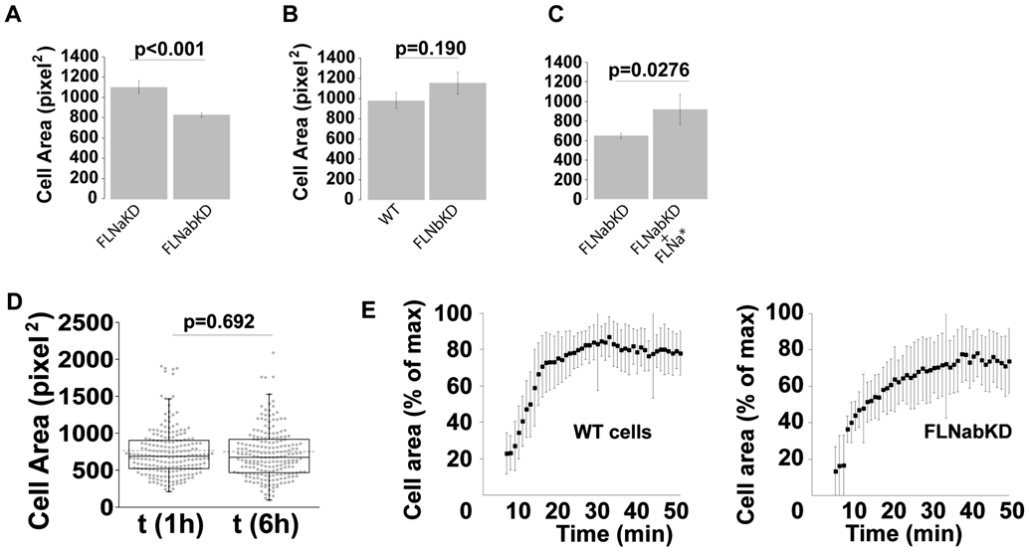
FLNabKD cells exhibit spreading defects. **A,B**) FLNaKD and FLNabKD (A) or HT1080 WT and FLNbKD (B) cells were mixed and plated on FN-coated plates. After 6 hours cells were fixed and stained with anti-FLNb to discriminate between the two populations. The area was measured by manually rendering the cell contour in the phase contrast. (Sample size: FLNaKD = 85; FLNabKD = 89 from 5 experiments; WT = 76; FLNbKD = 59 from 3 independent experiments) 1 pixel^2^ = 1.664 µm^2^. Error bars show SEM. **C**) FLNabKD cells were transfected with FLNa*-GFP. 24 hours later cells were detached, replated and allowed to adhere and spread as described in A and B. After fixation cells were stained with anti-FLNa to identify FLNa* expressing cells (sample size: ≥10 from 6 independent experiments). 1 pixel^2^ = 1.664 µm^2^. Error bars show SEM. **D**) Comparison of cell area at the start and end of time-lapse recording. 1 pixel^2^ = 1.664 µm^2^. Dot plot shows the overall population distribution (sample size = 213; from 10 independent experiments) dotted line shows the mean value, box and whiskers plots show quartiles. P values were calculated using a paired t-test. **E**) HT1080 WT and FLNabKD cells were plated on plates coated with 5 µg/ml FN, and analyzed in high resolution time-lapse (1 frame/minute) 5 minutes after the plating. Values are shown as the average % of the maximum cell area ±SEM (sample size: WT = 12; FLNabKD = 9 from 3 independent experiments).

We next investigated the actin cytoskeleton in spread FLNb and FLNab knockdown lines. As was the case for FLNa, the removal of FLNb in FLNbKD cells, did not dramatically impact the actin cytoskeleton (Figure 3D). Analysis of FLNabKD cells revealed a slight decrease in phalloidin staining (Figure 3D), suggesting a decrease in overall F-actin levels and western blotting showed that this was not related to a change in actin expression levels (Figure 3A). The FLNabKD cells present a normal morphology without plasma membrane blebbing (Figure 3D), however, as we have previously reported [27], removing both FLNa and FLNb reduces the final spread area of the cells (Figure 4A). Spreading was not affected in single FLNa or FLNb knockdown lines (Figure 1C and 4B). Expression of an shRNA resistant FLNa-GFP mutant (FLNa*) in FLNabKD cells is sufficient to completely rescue the cell spreading defect (Figure 4C) showing that the effect on spreading is due to reductions in FLN expression.

The reduction in area of FLNabKD cells cannot be explained by a delay in cell spreading as images from time-lapse migration assays showed that the cells were fully spread 1 hour after plating and did not spread further over an additional 5 hours (Figure 4D). Time-lapse analysis of early time points of cell spreading in WT and FLNabKD cells did however suggest a slight delay in the time needed to reach the maximal area as wild-type cell spreading appeared to plateau approximately 30 minutes post-plating while the FLNabKD cells plateaued around 40–45 minutes after plating (Figure 4E).

In summary, while knockdown of either FLNa or FLNb does not produce dramatic effects on HT1080 cell spreading or morphology, knockdown of both FLNa and FLNb impairs cell spreading and reduces F-actin levels without major effects on cell morphology.

### Knockdown of FLNa and FLNb impairs initiation of cell migration

**Figure 5:**
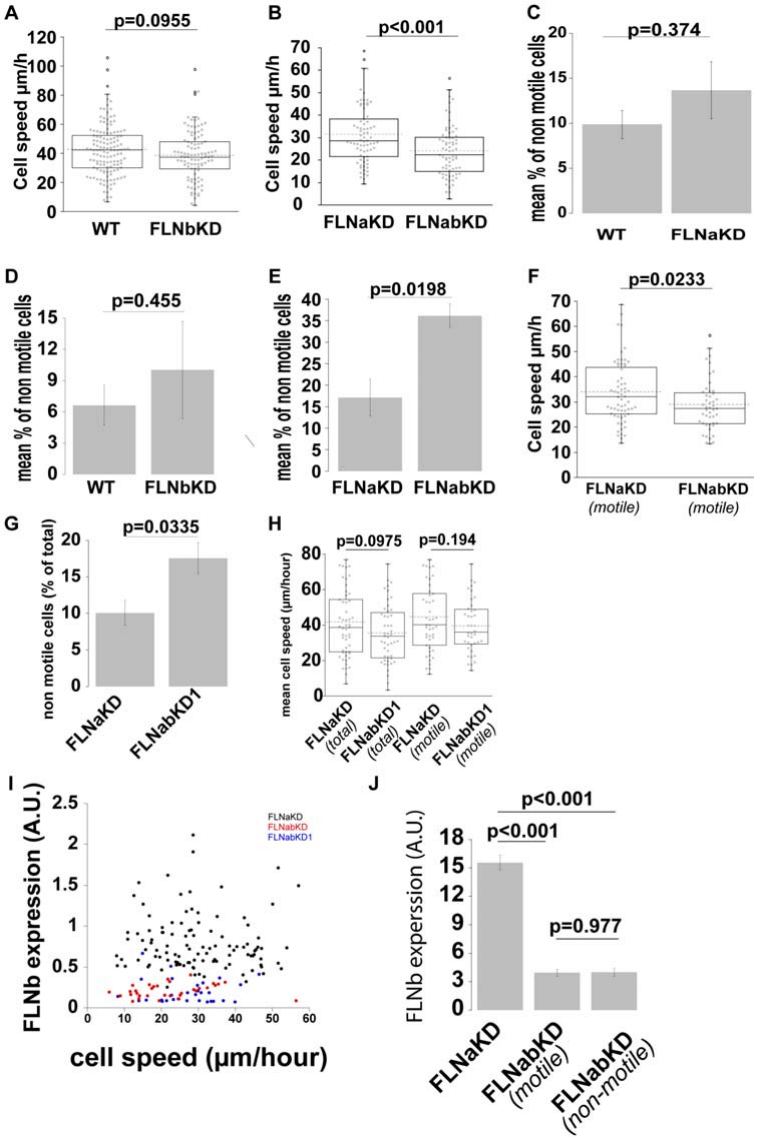
FLNs play a role in initiation of cell migration. **A,B,F** and **H**) Speed of HT1080 WT and FLNbKD (A), FLNaKD and FLNabKD (B), FLNaKD(motile) and FLNabKD(motile) (F), or FLNaKD and FLNabKD1 (H) were compared in time-lapse migration assays. Dot plot shows the overall population distribution (sample size: WT = 137; FLNbKD = 99; FLNaKD = 74 in B and 53 in H; FLNabKD = 71; FLNabKD1 = 48; at least 4 independent experiments were performed for each pair), dotted line shows the mean value, box and whiskers plots show quartiles. **C,D,E** and **G**) % of non motile cells in the time lapse experiments expressed as mean ± SEM. **I**) relation between cell speed and FLNb expression in FLNaKD (black dots), FLNabKD (red dots) and FLNabKD1 (blue dots). **J**) FLNb content of FLNaKD, FLNabKD motile and FLNabKD non-motile cells. Total fluorescence of FLNb staining was measured at the end of time-lapse recording. Bars show mean value ±SEM (sample size: FLNaKD = 58; FLNabKDmotile = 22; FLNabKDnon-motile = 15 from 3 independent experiments).

To assess the effect of FLNb knockdown on random cell migration we used time-lapse migration assays to compare wild-type and FLNbKD cells. Assays were performed as previously described (Figure 2A,B movie S2 and S3) and after fixation cells were stained for FLNb. Removal of FLNb did not significantly alter the speed of FLNbKD cells, although as was seen for FLNaKD cells, thereS was a trend towards a slight reduction in speed (Figure 5A; Figure 2D). However, comparison of the double knockdown cells (FLNabKD) with FLNaKD cells revealed a decrease in cell speed suggesting that removal of both FLNa and FLNb results in a migration defect (Figure 5B).

Careful examination of the time-lapse recording of FLNabKD cells suggested that many of these cells were not migratory at all. As described in the methods section, we track the movement of the nuclear region of the cell over time, and small oscillations in the position of the nucleus in non-migrating cells with active membranes, coupled with error associated with manual rendering of the nuclear position can result in calculation of low speeds for cells that are not actively migrating. Therefore, independently of cell speed, we scored cells as motile only if they had a maximum displacement of >25 µm, the average cell radius, at some point during the course of the assay. This means that to be scored as motile, irrespective of cell speed, the nucleus must exit a circle of radius 25 µm centered on the nuclear position in the first frame (Figure 2B). If the cell turns and subsequently re-enters the circle it will still be scored as motile because it had a displacement >25 µm at some point. This ensures that small oscillations do not result in a cell being classed as motile; instead motility requires a degree of persistent movement. Although the choice of one cell diameter is somewhat arbitrary, qualitatively our results do not change even if the threshold is reduced to 10 µm.

Scoring motility in our paired cell migration assays revealed that knockdown of FLNa or FLNb produced only small, not statistically significant, increases in the percentage of non-motile cells (Figure 5C–D). However, FLNabKD cells had a significant increase in non-motile cells compared to the FLNaKD line, and even after 5 hours of observation ∼35% of cells did not class as migratory (Figure 5E). This suggested that defects in initiation of migration might account for the reduced speed of FLNabKD cells. Indeed when the non-motile cells are excluded from the analysis the difference in speed between FLNaKD and FLNabKD cells is reduced in magnitude and significance (Figure 5F) but nonetheless a statistically significant reduction in speed remains.

**Figure 6:**
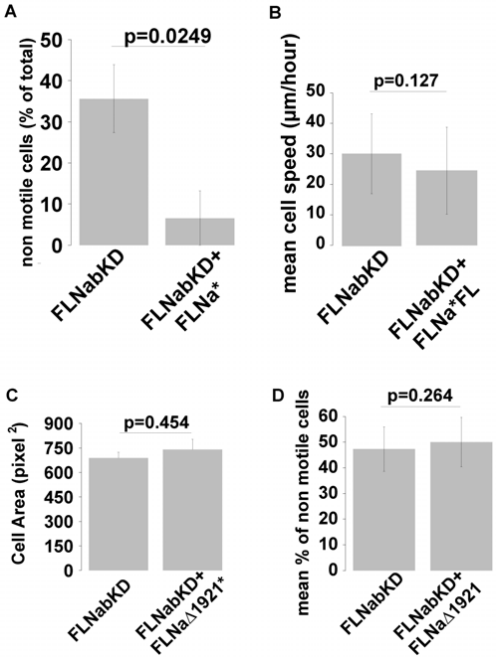
FLNa* but not FLNa*Δ19–21 rescues motility of FLNabKD cells. **A** and **B**) FLNabKD cells were transfected with FLNa*-GFP and assessed in time-lapse migration assays. Re-expressing cells were identified by staining for FLNa. Mean % of non-motile cells (A) and mean cell speed (B) in untransfected and FLNa-re-expressing FLNabKD cells. Values ±SEM from at least 4 independent experiments: sample size FLNabKD = 75; FLNabKD+FLNa* = 9). **C** and **D**) FLNabKD cells were transfected with FLNaΔ19–21 and assessed in time-lapse migration assays. Cells expressing FLNaΔ19–21 were identified by staining for FLNa. Mean cell area was measured by manually rendering the cell contour in the phase contrast (C) and motility assessed as described previously (D). Values ± SEM from at least 4 independent experiments: (sample size FLNabKD = 94; FLNabKD+FLNa*Δ19–21 = 10). 1 pixel^2^ = 1.664 µm^2^.

The data obtained with FLNabKD cells suggested that loss of FLNa and b impairs initiation of migration and may also reduce the mean speed of those cells that do migrate. As these conclusions were based on one double knockdown line we examined a second independent FLNab knockdown line (FLNabKD1). These cells expressed less that 10% FLNa and 35% FLNb compared to wild-type cells and had normal vinculin and actin levels (Figure 3A,B). Analysis of FLNabKD1 cells also revealed a significant increase in the percentage of non-motile cells (Figure 5G), but in this case the reduction in mean cell speed was not statistically significant (Figure 5H). Exclusion of non-motile cells from the analysis resulted in further convergence of mean speed of the motile FLNaKD and FLNabKD1 populations (Figure 5H). Comparison of FLNb expression with cell speed in FLNaKD, FLNabKD and FLNabKD1 cells (Figure 5I) shows that cell speed is independent of FLNb content. It is not clear why some FLNab knockdown cells are motile and some are not but differences in the degree of knockdown do not readily to account for this as comparison of FLNb levels between motile and non motile FLNabKD cells shows no significant difference (Figure 6J). As the FLNabKD cells were generated from the clonal FLNaKD line and this, like the FLNabKD line, shows high levels of stable FLNa knockdown we conclude that the motile cells do not have higher levels of FLN than the non-motile cells. We propose that FLNs regulate initiation of cell migration such that below a certain FLN expression threshold initiation is reduced and the probability of observing it during the time-lapse recording is decreased.

To further test whether the phenotypes observed in FLNabKD cells were specific and caused by loss of FLN expression, we attempted to rescue FLNabKD cells by re-expression of shRNA-resistant FLNa-GFP (FLNa*). This construct rescued the spreading defect in these cells showing that it is functional (Figure 4C). FLNabKD cells were transfected with FLNa* and migration was assessed in time-lapse assays as previously described. Cells were then fixed and stained for FLNa allowing us to quantify the levels of re-expressed FLNa. Cells expressing ∼1–2 fold wild-type levels of FLNa were scored for motility and speed and compared to non-expressing cells on the same plate. As shown in Figure 6A, re-expression of FLNa rescued the immotile phenotype of FLNabKD cells but did not increase the mean cell speed (Figure 6B) again suggesting that loss of FLN mainly effects initiation of cell migration rather than cell speed.

### IgFLNa domains 19–21 are required for FLNa-mediated cell spreading and initiation of cell migration

FLNs are important adaptor molecules and many filamin-binding proteins interact with IgFLNa domains 19 to 21 [5], [9]. As an additional control for the specificity of the FLNa rescue experiments, and to test whether IgFLNa19–21-mediated interactions may be involved in cell spreading and initiation of cell migration, we assessed the ability of an shRNA resistant FLNa-GFP mutant lacking IgFLNa19–21 (FLNaΔ19–21) to rescue the phenotype of FLNabKD cells. Unlike the wild-type FLNa-GFP protein, the FLNaΔ19-21-GFP protein did not rescue spreading (Figure 6C) or motility (Figure 6D) of FLNabKD cells, suggesting that deletion of IgFLNa19–21 produces a FLNa molecule that is defective in these processes, presumably because it is uncoupled from key pathways.

### HT1080 cells express Filamin C

**Figure 7:**
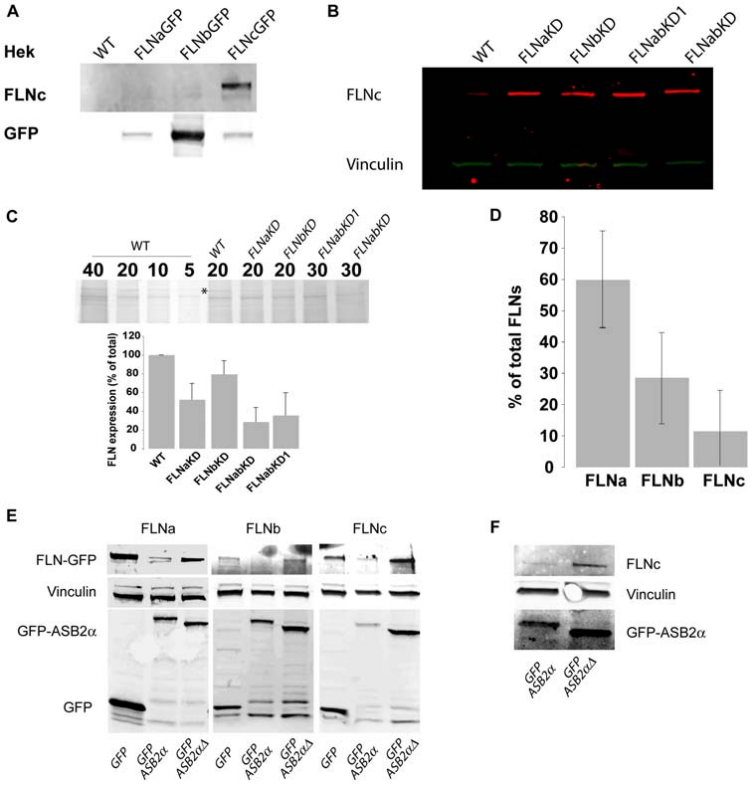
HT1080 express FLNc. **A**) Cell lysates from HEK 293 cells or HEK cells expressing FLNa-GFP, FLNb-GFP or FLNc-GFP were probed by an anti-FLNc antibody (upper panel) or an anti-GFP antibody (lower panel). **B**) 30 µg of lysates from the indicated cell lines were probed for FLNc content (red signal) and vinculin (green signal) used as loading control. **C**) Cell lysates were fractionated by SDS-PAGE and gels stained for total protein. The extent of staining of a band of approximately 280 kDa (marked with an asterisk) that co-migrates with bands detected by anti-FLNa antibodies was measured. Bars show the results from 5 experiments (mean ± SEM). **D**) Estimation of total FLN levels in HT1080 WT. **E**) HT1080 WT cells were co-transfected with FLNa-GFP (left panel), FLNb-GFP (middle panel), or FLNc-GFP (right panel) and GFP, GFP-ASB2α or GFP-ASB2αΔ as indicated. 24 hours after transfection cells were lysed, and analysed by western blotting for GFP. Vinculin staining was used as loading control. **F**) HT1080 FLNaKD cells were transfected with either GFP-ASB2α or GFP-ASB2αΔ. 20 hours after transfection cells were lysed and analysed by western blotting for FLNc and GFP. Vinculin was used as loading control.

FLNc has been reported to be largely specific to skeletal and cardiac muscles [6], [14] and no data are available on FLNc expression in fibrosarcoma cells. Using an antibody specific for FLNc (Figure 7A), we show that HT1080 cells express low, but detectable, levels of FLNc (Figure 7B). Moreover, FLNc expression is increased in the FLNa, FLNb and FLNab knockdown lines (Figure 7B). A pan-FLN antibody that binds each FLN isoform with equal affinity is not available therefore measuring total FLN levels in cells is not straightforward. However, when fractionated by SDS-PAGE all three FLNs migrate at approximately 280 kDa, in a region where there are few other proteins, meaning that a rough approximation of total FLN content can be obtained by protein staining. To estimate the extent of total FLN knockdown in the various cell lines, we fractionated cell lysates by SDS-PAGE and assessed the intensity of staining of a band of approximately 280 kDa that is selectively reduced in FLN knockdown lines and which co-migrates with bands detected by anti-FLNa antibodies (Figure 7C). Estimation of total FLN levels (Figure 7C), together with measurement of the extent of FLNa and FLNb knockdown, and FLNc over-expression in FLNaKD, FLNbKD and FLNabKD cells by western blotting (Figure 3A and Figure 7B), allowed us to approximate the relative amounts of each FLN isoform. In wild-type HT1080 cells FLNa represents 60%±15%; FLNb 29%±15% and FLNc 11%±13% of the total FLN (Figure 7D). We note that this analysis relies on the assumption that the entire ∼280 kDa band is composed only of FLNa, FLNb and FLNc while in fact it is possible that other large proteins contribute to this band. If this is the case, our calculation of total FLN knockdown in Figure 7C will be an underestimation (i.e. total FLN levels will be even lower in knockdown cells) and FLNc will make up a smaller percentage of the total FLN than indicated in Figure 7D. Thus, despite the large margin of error, this analysis shows that, total FLN levels are lower in the double knockdown FLNabKD cell lines than in the FLNaKD or FLNbKD lines even though these lines have increased levels of FLNc.

### ASB2α targets all 3 FLNs for degradation

**Figure 8:**
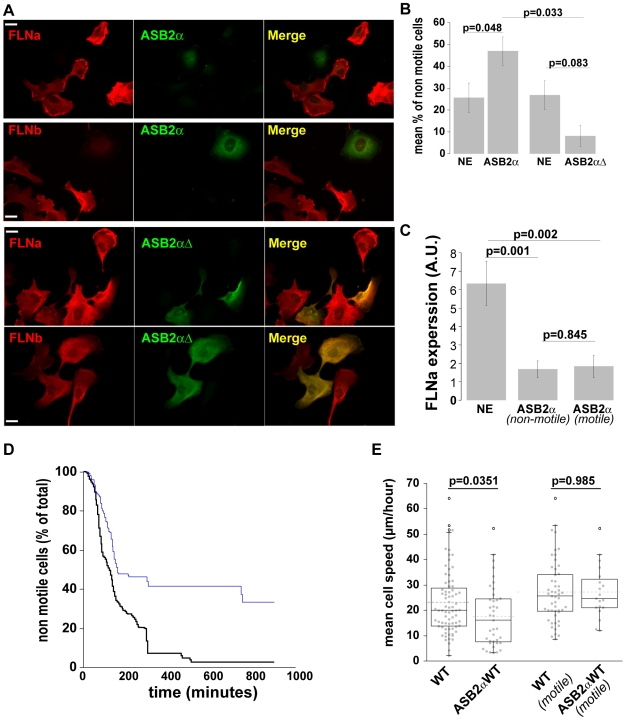
ASB2α inhibits initiation of cell migration without affecting cell speed. **A**) HT1080 transfected with GFP-ASB2α (left panel) or GFP-ASB2αΔ (right panel) were detached after 20 hours and fixed 6 hours after re-plating on 5 µg/ml FN and stained for FLNa or FLNb. **B**) Percent of non-motile cells in the time-lapse recording expressed as mean ± SEM (6 experiments for ASB2α; 3 for ASB2αΔ). **C**) HT1080 ASB2α expressing or non-expressing cells (NE) were stained at the end of time lapse experiments to measure FLNa content in motile and non motile cells (sample size NE = 20; ASB2α-motile = 12; ASB2α non motile = 17; from 3 independent experiments). **D**) HT1080 ASB2α expressing (blue line) or non-expressing (black line) cells were recorded for 15 hours at 1 frame/5 minutes and the mean percent of non motile cells at each time point plotted. (Sample size: NE = 43; ASB2α = 13; from 2 independent experiments). **E**) Speed of HT1080 ASB2α expressing or non-expressing cells: total population (left) or migratory population (right). (Sample size: NE all = 75; ASB2α all = 39; NE motile = 51; ASB2α motile = 19; from 6 independent experiments).

Identification of FLNc in HT1080 cells and its up-regulation in FLN knockdown cells complicates our analysis of the role of FLNs in cell migration. To circumvent generation of a triple knockdown line lacking FLNa, b and c, and avoid potential problems associated with clonal lines we used ASB2α, a component of an E3 ubiquitin ligase complex, to acutely trigger proteasomal degradation of all three FLNs. We have previously shown that ASB2α targets FLNa and FLNb for polyubiquitylation and proteasomal degradation [27], [28]. Furthermore, using a label-free quantitative proteomic strategy to identify ASB2α substrates [29] we observed that ASB2α expression also triggered degradation of the low levels of endogenous FLNc detectable in myeloid leukaemia cells (table S1). Consistent with these results, when co-expressed in HT1080 cells, ASB2α efficiently triggered degradation of FLNa-GFP, FLNb-GFP and FLNc-GFP (Figure 7E). Western blotting also shows degradation of endogenous FLNc after transient transfection with ASB2α (Figure 7F). In contrast, expression of an inactive ASB2α mutant (ASB2αΔ), which lacks the SOCS box rendering it unable to assemble into an E3 ubiquitin ligase complex [27], did not decrease FLN levels (Figure 7E,F and Figure 8A). Our anti-FLNc antibody does not work for immunofluorescence in HT1080 cells but staining with anti-FLNa and anti-FLNb antibodies shows the extent of FLNa and FLNb degradation in ASB2α-expressing HT1080 cells (Figure 8A). Thus, all three FLNs are ASB2α substrates and, as these are the only known ASB2α targets, transient transfection with ASB2α provides a mechanism to acutely degrade all three FLN isoforms and to generate FLN-deficient cells.

### Acute FLN-degradation impairs initiation of cell migration without altering cell speed

To test the effect of ASB2α-mediated FLN degradation on cell migration, HT1080 cells were transfected with ASB2α and the migration of ASB2α-expressing cells was compared to the cells in the same microscope field that were not expressing ASB2α and thus retained FLN expression (Figure 8A and movie S4). As an additional control we assessed the effect of ASB2αΔ, which did not degrade FLNs (Figure 8A), on migration. ASB2α expression significantly increased the percentage of non-motile cells (Figure 8B). The immotility phenotype is not due to ASB2α-induced toxicity as these cells attach in 10 minutes, spread within 1 hour, and remain attached and spread over the course of the 6 hour assay. To further test the hypothesis that loss of FLNs delays initiation of migration we extended our time-lapse assays to 16 hours and quantified the percentage of motile and non-motile cells in the ASB2α-expressing and non-expressing populations over the course of the experiment. The number of non-motile cells is higher in the ASB2α-expressing population at virtually all time points assessed (Figure 8D), however the percentage of non-motile cells decreases over time suggesting that cells eventually begin to migrate.

As was observed for FLNabKD cells, many ASB2α-expressing cells were capable of migrating. To determine whether differences in the efficiency of FLN degradation explained why some ASB2α-expressing cells were motile but not others, we assessed FLNa levels in the motile and non-motile ASB2α-expressing cells (Figure 8C). This clearly showed that FLNa was efficiently degraded in all ASB2α-expressing cells, whether motile or non-motile, suggesting that wild-type FLN levels are not essential for cell migration but instead, as described above, FLNs control initiation of cell migration

ASB2α expression also results in a reduction of mean cell speed (Figure 8E). However, this effect is primarily due to the delay in initiation of migration as when the non-motile cells are removed from the analysis the mean speed of the ASB2α-expressing and control cells is comparable. Taken together, the data from FLN knockdown cells and ASB2α-expressing cells, suggest that FLNs are important for initiation of cell movement, but that the presence or the absence of FLNs does not alter the speed of migrating cells.

### Knockdown of FLNa and FLNb impairs initiation of cell migration in Jurkat cells

**Figure 9:**
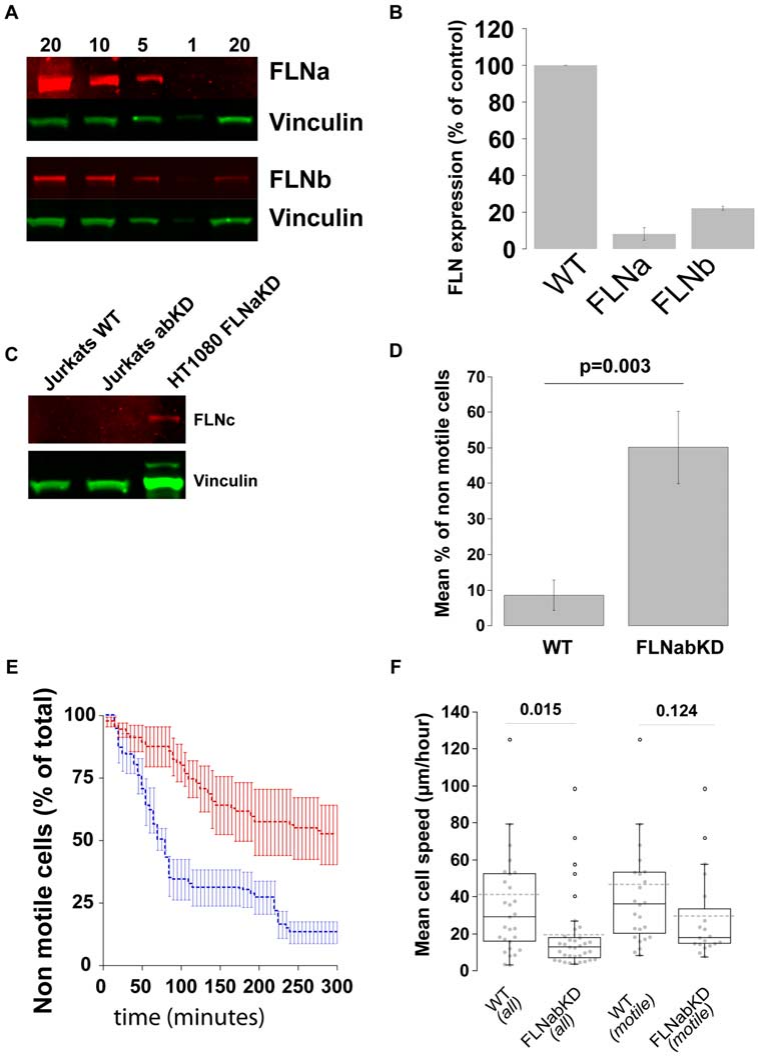
FLNs play a role in initiation of cell migration in Jurkat cells. **A**) FLNa and FLNb protein expression was quantified comparing 20 µg of cell lysate prepared from Jurkat FLNabKD cells to a curve of cell lysates prepared from Jurkat WT lysates. Vinculin was used as loading control. **B**) Quantification of FLNa and FLNb protein expression; bars show mean value normalized to control ±SEM (n = 4). **C**) 20 µg of a cell lysate from Jurkat WT or Jurkat FLNabKD was probed for FLNc content, 20 µg of lysate from HT1080 FLNabKD cells was used as positive control. **D**) Percent of non-motile cells in the time-lapse recording expressed as mean ± SEM (from 6 independent experiments). **E**) Percent of non-motile cells were analysed at each time point in 5 experiments and the average ± SEM is plotted. Blue line shows Jurkat WT cells red line Jurkat FLNabKD cells. **F**) Speed of Jurkat WT and FLNabKD cells: total population (left) or migratory population (right). (Sample size: WT all = 29; FLNabKD all = 35; WT motile = 25; FLNabKD motile = 19; from 6 independent experiments).

The preceding sections show that in HT1080 cells knockdown or proteolytic degradation of FLNs impairs the ability of cells to become motile. To test whether this is a more general phenomenon we analysed the migration of the Jurkat immortalized T lymphoblast cell line. FLNabKD Jurkat cells stably transfected with shRNA against FLNa and FLNb were generated and showed dramatically reduced levels of FLNa and FLNb (Fig. 9 A and B) with no detectable expression of FLNc (Fig. 9 C). FLN knockdown did not impact β1 integrin expression levels in these cells (data not shown). Jurkat cells are smaller and more rounded than HT1080 cells which facilitates the scoring of cells as motile or non-motile (see movie S5). As observed with HT1080 cells, the FLNabKD Jurkat cells show a significant increase in the percentage of non-motile cells (Fig. 9D). Furthermore, similar to ASB2α-transfected cells (Fig. 8 D) the percentage of non motile cells decreases over time for both wild-type and FLNabKD cells but the percentage of non-motile cells remains higher in FLNabKD cells at virtually all time points (Fig. 9E). The decrease in non-motile cells over time indicates that cells initially scored as non-motile can initiate movement showing that non-motile cells are not dying and that lack of FLNs does not completely block the initiation of migration. Finally FLNabKD Jurkat cells exhibit a decrease in average speed which is, as in the case of ASB2α transfected cells, primarily due to the increased presence of non-migratory cells in the overall population (Fig. 9F left panel). In fact, removing the non-motile cells from the analysis decreases the difference of mean cell speed under the level of statistical significance (Fig. 9F right panel). Thus the effect of FLN-deficiency on cell motility is conserved in both HT1080 fibrosarcoma cells and Jurkat T-lymphoblasts.

## Discussion

A role for FLNs in cell migration was first proposed 17 years ago based on the phenotype of FLNa-deficient melanoma cells [15]. The idea that FLNa is important for migration was reinforced by identification of loss-of-function mutations in FLNa as causative for PVH [16], a disease associated with defective neuronal migration. However, the identification of FLNb and FLNc [11]–[13] and the finding that FLNa and FLNb are co-expressed in some cell types [25] raised the potential that other FLN isoforms may also function in migration. More recent data from FLNa-deficient mice have questioned the importance of FLNa in cell migration [19], [20] however we note that while FLNa-deficient mice do not develop PVH they do exhibit a thinning of the cerebral cortex that may indicate a role in migration [20]. In order to address the question of whether FLNa, and/or other FLNs, play roles in migration, and if so to investigate at which stages they are important, we used shRNA-mediated knockdown and ASB2α-mediated proteasomal degradation as two independent methods to remove FLNs from cells. We found that almost total ablation of either FLNa or FLNb does not significantly affect the ability of HT1080 cells to migrate in time-lapse random migration assays but that shRNA-mediated knockdown of both FLNa and FLNb, or ASB2α-mediated targeting of all three FLNs, leads to defects in migration. This establishes a role for FLNs in migration and suggests that compensation by other FLN isoforms [25] may mask the migration phenotype in single knockdown cells or single knockout cells or animals. We further characterised the migration defect to be primarily a deficiency in initiation of motility rather than a problem with maintenance of locomotion speed once migration has been initiated. In addition, while we observe no profound impact of FLN deficiency on cell shape or the actin cytoskeleton, we find that FLN-deficient cells spread to a lesser extent than wild-type or single knockdown cells. Thus FLNs are important for cell spreading and contribute to the initiation of cell migration, but appear dispensable for maintenance of normal cell speed once migration has been initiated.

Much of the work implicating FLNa in cell migration has relied on use of a FLNa-deficient melanoma cell line (M2) and stable cell lines re-expressing FLNa [15]. While the M2 line has proved valuable, differences in expression of cell surface proteins, such as integrin adhesion receptors, between the M2 and reconstituted lines [30] complicates its use in migration assays. For this reason we used alternative methods to generate FLN-deficient HT1080 cells that did not show alteration in integrin expression levels. Use of both stable knockdown lines and acute ASB2α-mediated degradation provides a number of advantages. Knockdown lines offer the convenience of stable populations and the ability to rescue phenotypes by re-expressing knockdown-resistant FLN confirms the specificity of observed phenotypes. However, while knockdown was extensive it was not complete and FLNc levels were elevated. We therefore also used transient ASB2α expression to acutely remove all three FLNs. An additional benefit of this approach is that since FLNs are rapidly degraded the risk of selecting cells with compensatory adaptations that mitigate the effects of loss of FLNs is greatly reduced. Unfortunately, due to the number of lysines in these large proteins (156 in FLNa and 173 in FLNb) and the lack of a detailed understanding of the mechanism by which ASB2α targets FLNs for degradation, we cannot yet generate ASB2α-resistant FLNs for rescue experiments. Hence, we cannot completely exclude effects of other yet-to-be-identified ASB2α targets on cell migration. However, the similarity in phenotype of ASB2α-expressing and FLNabKD cells points to the importance of FLNs during initiation of migration and cell spreading. Furthermore the similarity in phenotype between FLN-deficient HT1080 and Jurkat cells suggests that FLNs have a general role in migration that is not restricted to a single cell type.

Our time-lapse migration assays allowed us to observe that the major defect associated with loss of FLNa and FLNb was failure to initiate migration rather than a change in mean speed of migration or directional persistence. Both ASB2α expression and knockdown of FLNa and FLNb resulted in significant increases in the percentage of cells that failed to migrate during the experiment and the phenotype was reversed by re-expressing FLNa in the FLNabKD cells. In FLNaKD cells the number of non-motile cells also consistently increased but this did not reach statistical significance in the paired experiments (n = 38 in WT and 41 in FLNaKD). However, combining data from all the HT1080 WT and FLNaKD experiments (even those not directly comparing WT and FLNaKD on the same plate) revealed a small but statistically significant increase in the percentage of non-motile FLNaKD cells (WT 7.3% n = 175; FLNaKD 15.7% n = 116 p = 0.01). This suggests that loss of 95% of FLNa expression is sufficient to produce a mild migration phenotype but that the additional loss of FLNb is required to produce a more robust effect, presumably because FLNb compensates for the lack of FLNa [25]. Even in FLNabKD or ASB2α-expressing cells the penetrance of the inhibition of motility was not complete and many knockdown cells did migrate, furthermore the number of motile cells increased over time; suggesting that FLNs are not indispensable for initiation of migration but that they control the propensity that a cell has to initiate migration. Thus while FLN-deficient cells are capable of migration in any given time period they are less likely to initiate migration. One possible explanation for this is that the initiation of migration is controlled both by internal and external signals and that loss of FLN can be overcome by external stimuli such as polarization due to random asymmetry in the coating surface.

We note that in our assay the definition of motility requires a minimum displacement from the origin and accept that the threshold at which we consider cells motile is somewhat arbitrary. We have selected a nuclear centroid displacement of approximately one cell diameter to constitute a motile cell, as cells showing this degree of displacement are clearly motile. However, use of a smaller threshold yields a very similar conclusion, namely that loss of FLN increases the percentage of non-motile cells. Our assay is not dependent on cell speed and so helps us to discriminate between truly motile cells and immotile cells that oscillate around their origin due to small changes in nuclear position or error in manual rendering of the nucleus on recorded images. Drawbacks of this method are that very slowly moving cells may be scored as non-motile because they fail to move far enough in the 5 h recording and that once a cell starts to migrate it take some time, depending on cell speed and persistence, to pass the displacement threshold and class as motile so there will be a lag in classifying cells, particularly slower cells, as motile. Overall we do not think that these present major problems for the interpretation of our data because as mentioned previously the results hold true for a range of cell types using different displacement thresholds and because we have based most of our conclusions on results obtained after 5 hours of migration.

It is well established that FLNs are important actin crosslinking proteins [5] and FLNa stabilizes the orthogonal actin networks in membrane protrusions [26]. Given the role of FLNs in actin crosslinking it is somewhat surprising that no major effects on F-actin staining or shape of FLN knockdown, or ASB2α-expressing, HT1080 cells were evident. However, FLNa knockout cells also retained the ability to organise the actin cytoskeleton [19], [20] and stress fibres were largely unchanged in other ASB2α-expressing lines [27] indicating that this is not an HT1080 specific effect. We note that our knockdown lines are not fully FLN-deficient and residual FLNa or FLNb along with up-regulated FLNc may provide sufficient crosslinking activity so we do not exclude a role for FLNs in regulation of the actin cytoskeleton. Indeed, double knockdown FLNabKD cells show somewhat reduced F-actin staining and in single FLNaKD cells stress fibres were thinner than those in wild-type cells (Baldassarre and Calderwood unpublished data). Loss of both FLNa and FLNb did however result in cells that spread less well than wild-type cells and like initiation of cell migration this required the IgFLNa19–21 domains suggesting that the spreading and initiation of migration phenotypes may be linked.

Currently we favour the idea that FLNs enhance initiation of migration through interactions with associated signalling or receptor molecules that govern early steps such as polarization, membrane dynamics, contractility or symmetry breaking [5], [23], [31], [32]. In support of this idea, re-expression of full-length FLNa rescues the defect in initiation of migration in FLNabKD cells, but the phenotype is not rescued by the FLNaΔ19–21 mutant, which should still be capable of binding and crosslinking F-actin [33]. This suggests that some of the more than 20 reported FLN-binding proteins whose interactions sites overlap with IgFLNa19–21, may contribute to initiation of migration. Future work will seek to identify which of these are important. Notable candidates include integrin [34], migfilin [35] and Rho family GTPases [5]. However, as IgFLN domains can form domain-pairs and so regulate neighbouring domains [36] the Δ19–21 mutation could also exert its effects by modulating ligand binding to adjacent IgFLN domains.

Although we detect clear effects of FLN-deficiency on initiation of migration we do not find an evident role for FLNs in control of cell speed as there was no consistent difference in the speed of motile FLN expressing and deficient cells. We conclude that FLNs are not important for determining cell speed but accept that low levels of residual FLN in our cells means that we cannot completely rule out a role for FLN in setting cell speed.

Our data provide an explanation for the apparently conflicting results in the literature. Compensation by other FLN isoforms is a likely explanation for the lack of major migratory defects in most tissues of single knockout animals. Furthermore, as loss of FLNs results in a defect, or delay, in initiation of migration rather than a complete block, it might be predicted that tissues in which the exact timing of migration is critical will be the most severely effected by loss of FLNs. This appears to be the case in human PVH patients.

## Materials and Methods

### Reagents and DNA constructs

Monoclonal anti-FLNa (Chemicon), monoclonal anti-vinculin antibody (Sigma), monoclonal anti-Actin (Abcam) polyclonal anti-GFP (Rockland), polyclonal anti-FLNc (Kinasource), anti-CD29 (Dako, Via Real Carpinteria CA), secondary anti-rabbit Alexa-568, anti-rabbit and anti-goat Alexa-680 conjugated (Invitrogen, Carlsbad CA), anti-mouse FITC conjugated (Pierce, Rockford, IL ), anti-mouse IRDye 800 (LI-COR Biotechnology, Lincoln NB), phalloidin alexa-568 conjugated were purchased. Anti-FLNa and anti-FLNb anti serum raised against the domains 19 to 21 of the respective proteins and were previously described [27]. Fibronectin (FN) solution 1 mg/ml was purchased from Sigma. FLNa-GFP and FLNaΔ19–21 have been described previously [34], [36], FLNb-GFP was provided by A. Sonnenberg (Netherlands Cancer Centre, Netherlands), [37] and FLNc-GFP was a gift from D. O. Fürst and P. F. M. van der Ven (University of Bonn, Germany). GFP-tagged knockdown resistant FLNa*GFP and FLNa*Δ19–21 were generated by QuikChange site-directed mutagenesis kit (Stratagene) and confirmed by DNA sequencing. FLN-targeting shRNAs in the pSM2c and pGIPZ vectors were purchased from OpenBiosystems. GFP-ASB2α and GFP-ASB2Δ expression vectors were described previously [27].

### Cell Culture

Human fibrosarcoma HT1080 cells were cultured in Dulbecco's Modified Essential Medium (Invitrogen) containing 9% fetal bovine serum (Atlanta Biological, Lawrencewille, GA) and penicillin/streptomycin (Invitrogen) and incubated at 37 °C in a humidified atmosphere containing 5% CO_2_. Jurkat cells were cultured in RPMI containing 9% fetal bovine serum, and penicillin/streptomycin and incubated at 37°C in a humidified atmosphere containing 5% CO_2_. For transfection, HT1080 cells were seeded at 50% confluence and transfected 24 hours after plating using Lipofectamine 2000 (Invitrogen) or seeded at 70% confluence and transfected 8 hours later using calcium phosphate protocols [38]. Jurkat cells in exponential growth phase were electroporated using a BioRad Genepulser II and Ingenio Electroporation Solution (Mirus Bio LLC) according to the manufacturers' instructions.

### Immunofluorescence

Cells, seeded on either FN-coated coverslips or 3.5 cm Petri dishes (Becton Dickinson), were fixed in 4% paraformaldehyde in phosphate buffered saline (PBS) pH 7.4 for 15 minutes and permeabilized for 30 min with PBS containing 0.02% saponin, 0.2% bovine serum albumin (BSA) and 50 mM NH_4_Cl. Cells were then incubated with primary antibodies of interest or fluorophore-conjugated phalloidin for 1 hour at room temperature, then, when necessary, incubated with fluorophore-conjugated secondary antibodies for 45 minutes washed again in PBS and coverslips were mounted using the ProLongGold anti-fade mounting agent (Invitrogen). Images were acquired on a Nikon TE2000 with a 10× or 40× objective using IPLab (version 3.5.2; Scanalytics, Fairfax VA) software and analyzed using ImageJ (U. S. National Institutes of Health, Bethesda, MD, http://rsb.info.nih.gov/ij/; versions 1.38–1.42).

### Immunoblotting

Cells were lysed in RIPA buffer (0.35 M Tris pH 7.2, 0.5 M NaCl, 10 mM MgCl_2_, 1% Triton X-100, 0.1% SDS, 0.5% Sodium Deoxicolate) containing Protease Inhibitors Cocktail Tablets (Roche). Lysates were run on SDS-PAGE, transferred onto nitrocellulose membrane, blocked for 1 hour with 5% not-fat milk in T-TBS (0.1 M Tris pH 7.4, 135 mM NaCl, 0.05% Tween-20). Membranes were probed 1 hour at room temperature or overnight at 4°C with primary antibody, washed in T-TBS and incubated for 1 hour with fluorescent secondary antibodies. Signal was detected using the Odyssey infrared imaging system (LI-COR Biotechnology).

### Generation of FLN knockdown cell lines

Polyclonal HT1080 FLNa knockdown cell lines were generated by transfecting HT-1080 wild-type (WT) cells with pSM2 vector expressing FLNa shRNA and selected using 2 µg/ml Puromycin (Sigma). Single clone lines were obtained by limiting dilution of the polyclonal population. Single clones were screened for FLNa content by immunofluorescence staining with FLN antibodies in 96 well plates and the signal quantified on a fluorescence plate reader (Safire). To correct for well to well variation in cell number the FLN signal was normalized to actin. FLNb and FLNab knockdown cell lines were generated by transfecting HT-1080 WT and single cloned FLNaKD with pGIPZ vector expressing FLNb shRNA. Polyclonal populations were selected using 1 mg/ml Hygromycin (Invitrogen). Jurkat FLNa and FLNab knockdown were generated as above with the following modification; since the pGIPZ vector codes for GFP in addition to the shRNA cassette, after establishing a polyclonal FLNabKD line cells were FACSorted to select a homogeneous GFP-expressing population allowing enrichment of knockdown cells.

### Quantification of FLN expression levels

To quantify FLN expression in the knockdown cell lines, increasing amounts of lysate from WT cells were run alongside knockdown lysates after western blotting probed with anti-FLNa or anti-FLNb antibodies and anti-vinculin and anti-actin antibodies as loading controls. Membranes were scanned using the Odyssey infrared imaging system. The signals were quantified using ImageJ, and FLN knockdown calculated according to the standard curve generated from the WT lysates after correction for loading using the actin and vinculin signals.

### Cytofluorometry

Cells were detached in trypsin EDTA, washed twice in PBS, then incubated for 30 min at 0°C with anti-CD29 (integrin Beta1), washed twice in PBS, incubated other 30 min at 0°C with anti-mouse FITC conjugated, washed twice in PBS and analysed using FACS Calibur machine (BD Bioscience).

### Time-lapse migration assays

Cells were washed, detached and plated on 5 µg/ml (FN) coated non-tissue culture treated 3.5 cm dishes. Grids were etched on the bottom of the plate to aid location of cells following staining. Both control and knockdown cells were seeded in the same dish. Ten minutes after plating, unattached cells were removed by washing. Starting 1 hour after plating cells are imaged by phase contrast every 5 min for 5 hours, or for 15 hours, on a Nikon TE2000 with a 10× objective. The cells were kept at 37°C using a home-designed thermostatic chamber. Cells were then fixed with 4% PFA for 15 min and immuno-fluorescence staining for FLNa or b was performed. Cells in the imaged field are identified and FLN levels were measured using ImageJ by multiplying the mean fluorescence, corrected for the background, by the area of the cell.

Jurkat cells were analysed during their exponential growth phase. A mix of WT and FLNabKD cells were plated on 3.5 cm dishes coated with 10 µg/ml FN and washed 30 minutes after plating then processed as above.

Analysis of the track of each cell in the field is quantified by manual rendering of the nuclear profile in all frames allowing localization of the coordinates of the nucleus centroid and calculation of path length, displacement from origin, and average speed (total path length/time). To allow rapid classification based on these parameters, special macros were developed in the open source software ImageJ. Cells entering in the field, exiting the field or those that divide during the time-lapse recording were excluded from the analysis. Error associated with manual rendering of images at 10× can result in low speeds (up to 5 µm/hours) in otherwise stationary cells. Thus in both knockdown and ASB2α transfection experiments, cells were scored as “motile” if the maximum displacement from the origin was ≥25 µm in HT1080 and ≥9 µm in Jurkat cells (∼cell radius). Cell area and circularity were measured by manually rendering the cells in the phase images. Graphing and statistical analyses were performed using KaleidaGraph 4.02 (Synergy Software, Reading, PA). All p values were calculated using t-test (populations were of equal variance) or, where indicated, paired t-test.

## Supporting Information

Table S1 Identification and label-free quantification of FLNa, FLNb, FLNc and Talin 1 in myeloid leukaemia cells expressing wild-type or an E3 ubiquitin-ligase defective mutant of ASB2
(0.04 MB DOC)

Movie S1 Cells were suspended, washed, and HT1080 WT and FLNaKD mixed before seeding onto 5 µg/ml FN-coated plates, 10 minutes after plating unattached cells were washed away and imaging was initiated 1 hour after plating at 1 frame/5 minutes for 5 hours. At the end of the time-lapse recording, cells were fixed and stained for FLNa expression. FLNaKD cells were outlined in the last frame and in the immunofluorescence image. Bar  = 20 µm.
(0.90 MB MOV)

Movie S2 Cells were suspended, washed, and HT1080 WT and FLNbKD mixed before seeding onto 5 µg/ml FN-coated plates, 10 minutes after plating unattached cells were washed away and imaging was initiated 1 hour after plating at 1 frame/5 minutes. At the end of the time-lapse recording, cells were fixed and stained for FLNb expression. FLNbKD cells were outlined in the last frame and in the immunofluorescence image. Bar  = 20 µm
(0.86 MB MOV)

Movie S3 Cells were suspended, washed, and HT1080 FLNaKD and FLNabKD mixed before seeding onto 5 µg/ml FN-coated plates, 10 minutes after plating unattached cells were washed away and imaging was initiated 1 h after plating at 1 frame/5 minutes for 5 hours. At the end of the time-lapse recording, cells were fixed and stained for FLNb expression. FLNabKD cells were outlined in the last frame and in the immunofluorescence image. Bar  = 20 µm.
(0.80 MB MOV)

Movie S4 HT1080 cells were transfected with ASB2α. After 20 hours cells were suspended, washed, and seeded onto 5 µg/ml FN-coated plates, 10 minutes after plating unattached cells were washed away and imaging was initiated 1 hour after plating at 1 frame/5 minutes for 5 hours. At the end of the time-lapse recording, cells were fixed and stained for FLNa expression. ASB2α expressing cells were outlined in the last frame and in the immunofluorescence image. Bar  = 20 µm.
(0.96 MB MOV)

Movie S5 WT and FLNabKD Jurkat cells were mixed before seeding onto 10 µg/ml FN-coated plates, 20 minutes after plating unattached cells were washed away and imaging was initiated 1 h after plating at 1 frame/5 minutes for 5 hours. At the end of the time-lapse recording cells were imaged in fluorescence to discriminate FLNabKD (expressing GFP) from WT cells. FLNabKD cells were outlined in the last frame Bar  = 20 µm..
(0.12 MB MOV)
