# Supplementary material for: Filamins Regulate Cell Spreading and Initiation of Cell Migration
Source: PLoS One. 2009 Nov 13;4(11):e7830. doi: 10.1371/journal.pone.0007830 (PMC2773003; doi:10.1371/journal.pone.0007830)
Supplement: Table S1 — Identification and label-free quantification of FLNa, FLNb, FLNc and Talin 1 in myeloid leukaemia cells expressing wild-type or an E3 ubiquitin-ligase defective mutant of ASB2 (0.04 MB DOC) [file pone.0007830.s001.doc]

**Table S1.** **Identification and label-free quantification of FLNa, FLNb, FLNc and Talin 1 in myeloid leukaemia cells expressing wild-type or an E3 ubiquitin-ligase defective mutant of ASB2α.** FLNa, FLNb, FLNc and Talin 1 were identified by shotgun proteomic analysis in cytosolic extracts of myeloid leukemia cells expressing wild-type ASB2α (wt) or the ASB2αLA mutant (LA). Relative label-free quantification of these proteins was performed by spectral count. One representative experiment out of 2 is shown. NA, not applicable.

^a^Mascot protein score obtained from the slice where the protein was identified with the best score.

^b^Corresponding sequence coverage.

^c^Number of total spectrum-to-peptide matches for all peptides specific to indicated proteins.

^*^From Burande CF, et al. 2009.

|  |  |  | identification | | | | quantification | | | | |
| --- | --- | --- | --- | --- | --- | --- | --- | --- | --- | --- | --- |
| Protein | Swiss-Prot acc. No. | Molecular mass | Mascot ^(a)^ protein score | | Sequence ^(b)^ coverage (%) | | Specific peptides | | Spectral ^(c)^ count | | Spectral count ratio |
|  | | | WT | LA | WT | LA | WT | LA | WT | LA | LA/WT |
| *FLNa | P21333 | 280564 | 1270 | 5941 | 28.8 | 66 | 49 | 502 | 79 | 893 | 11.3 |
| *FLNa | O75369 | 278021 | 107 | 417 | 1.8 | 13.9 | 0 | 33 | 0 | 43 | NA |
| FLNc | Q14315 | 290778 | - | 115 | - | 5.4 | 0 | 20 | 0 | 23 | NA |
| *Talin 1 | Q9Y490 | 269599 | 8232 | 6908 | 69.9 | 61.8 | 604 | 697 | 1146 | 1265 | 1.1 |
